# Comorbidity of intestinal helminthiases among malaria outpatients of Wondo Genet health centers, southern Ethiopia: implications for integrated control

**DOI:** 10.1186/s12879-019-4290-y

**Published:** 2019-07-24

**Authors:** Nigatu Tuasha, Elifaged Hailemeskel, Berhanu Erko, Beyene Petros

**Affiliations:** 1 College of Natural and Computational Sciences, Mizan-Tepi University, P.O. Box 121, Tepi, Ethiopia; 2 College of Natural and Computational Sciences, Wollo University, P.O. Box 1145, Dessie, Ethiopia; 3 Aklilu Lemma Institute of Pathobiology, Addis Ababa University, P. O. Box, 1176 Addis Ababa, Ethiopia; 4 Department of Microbial, Cellular and Molecular Biology, College of Natural Sciences, Addis Ababa University, P. O. Box, 1176 Addis Ababa, Ethiopia

**Keywords:** Coinfection, Helminthiases, Malaria, Wondo Genet, Ethiopia

## Abstract

**Background:**

It is estimated that over a third of the world population is infected by malaria and helminthiases mainly among communities with high poverty indices. The distribution of these parasitic infections overlaps in many epidemiological settings and have varying outcomes in the host. In this paper we report the prevalence of malaria and intestinal helminthiases coinfections among malaria suspected patients and the association of helminthiases with the occurrence of malaria and its outcomes in Wondo Genet, southern Ethiopia.

**Methods:**

In a cross-sectional study conducted from December 2009 to July 2010 in Kella, Aruma and Busa Health Centers in Wondo Genet, a total of 427 consenting febrile patients were screened for malaria and intestinal helminths infections. Malaria parasite detection and quantification were done using Giemsa stained thick and thin blood films. Helminth infections were screened and quantified by Kato-Katz thick smear method. Haemoglobin level was assessed using haemocue machine (HemoCue HB 201^+^). Difference in proportions and means were tested by Student’s *t* test and ANOVA while logistic regression analysis was used to determine the association between variables.

**Results:**

Of the total examined, 196 (45.90%) were positive for at least one helminth infection while 276 (64.64%) were positive for malaria. The prevalence of *Plasmodium falciparum* and *P. vivax* infections were 47.31 and 16.62%, respectively. The most common helminth parasites detected were *Ascaris lumbricoides* (33.96%), *Trichuris trichiura* (21.55%), *Schistosoma mansoni* (13.35%), and hookworms (6.79%). The overall malaria-helminthiases coinfection was 33.96%. The prevalence of anaemia was 43.12%. Helminthiases coinfection showed a positive correlation with the occurrence of malaria (AOR = 2.17, 95% CI: 1.44–3.28; *P* < 0.001). *Schistosoma mansoni* coinfection was associated with the increased risk of developing malaria associated anaemia (OR = 14.4, 95% CI: 1.37–150.80; *P* = 0.026).

**Conclusion:**

Malaria and helminth coinfections are important causes of morbidities among the population in Wondo Genet necessitating integrated control measures. Nevertheless, further detailed studies on the consequences and pathogenesis of these coinfections are needed to institute sound control and intervention measures.

## Background

Malaria and intestinal helminths are co-existing important public health problems in many parts of sub-Saharan African [1, 2]. According to World Health Organization (WHO), there were an estimated 216 million cases of malaria in 91 countries and increase of 5 million cases over the year 2015. Global malaria death was 445, 000 in 2016, which showed a reduction of 37% when compared to that in 2010. However, 90% of all global malaria cases and 91% of deaths are still shared in the WHO African Region alone [3].

Helminth infections are the most prevalent of chronic human infections and based on available estimates there are 1,221 million (*A. lumbricoides*)*,* 795 million (*T. trichiura*), and 740 million (hookworms) infections [2]. Schistosomiasis transmission is reported in 78 countries with 206.4 million people required preventive treatment in 2016 and of which 91.4% live in Africa [4]. Globally, different forms of coinfections exist, mainly among communities with high poverty indices. It is estimated that over a third of the world’s population is infected by malaria and parasitic helminths largely in tropical and subtropical regions [2, 5].

Although, often unclear to predict how and when the coinfections matter the most, accumulating evidence indicates that the course of the disease and outcome of the interventions are affected [6]. Malaria and intestinal helminthiases coinfections overlap in many epidemiological settings [5, 7, 8]. Such coinfections have varying outcomes in the host. Several reports showed an increase in malaria incidence associated with one or more intestinal helminths [9–13]. The risk of anaemia in patients might be increased with coinfection of helminths and malaria parasites [14–17]. On the other hand, helminths were observed to be protective of acute renal failure and jaundice [18] and cerebral malaria [19] among coinfected malaria patients. Some authors have also found that helminths - malaria coinfections have little or no significant contribution to the severity and parasite clearance rate of malaria [8] as well as on the incidence of malaria [20].

A detailed knowledge of intestinal helminths and malaria coinfection in endemic areas is very crucial to institute better prevention and control measures. Therefore, the objectives of this study were to assess the prevalence of malaria and intestinal helminthiases coinfection among malaria suspected patients as well as to determine the association of helminthiases with the occurrence of malaria and its outcomes in Wondo Genet, Sidama Zone, southern Ethiopia.

## Methods

### The study area

**Fig. 1:**
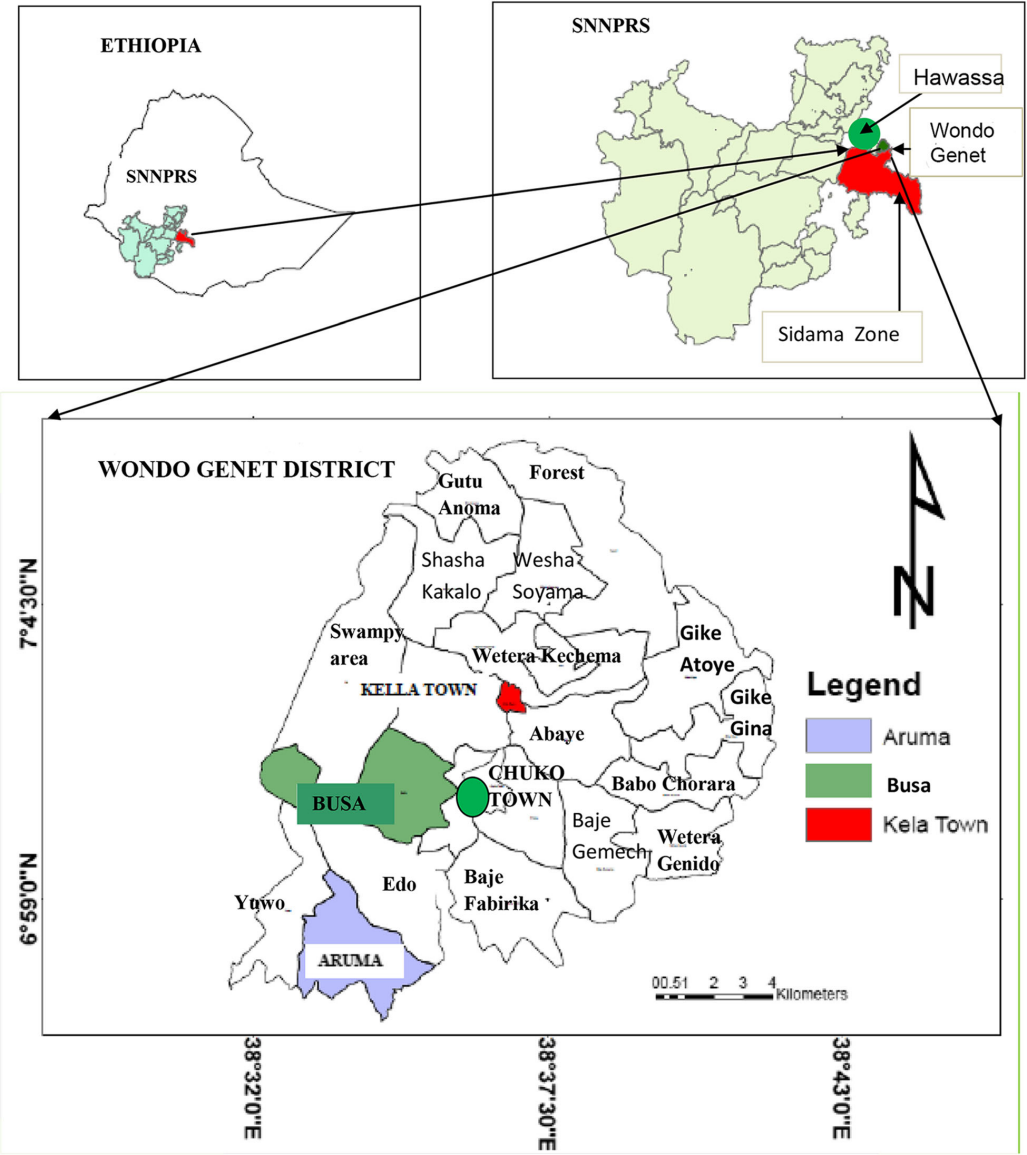
Map* showing the study centers and the surrounds, Wondo Genet District, Sidama Zone, SNNPRS, Ethiopia. *Note: Hawassa is transliterated as “Hawasa”, “Awassa” or “Awasa”; and Wondo Genet is also transliterated as “Wendo Genet”. Aruma and Kella Health Centers are located in the respective ‘*kebeles’*. Whereas Busa is a clinic in Chuko town (indicated with green spot) bordering Bula/Busa *‘kebele’*

The study was conducted in Wondo Genet (Fig. 1) which is about 270 km to the south of Addis Ababa and 24 km east of Hawassa City. Wondo Genet is one of the resort towns located in Sidama Zone, Southern Nations, Nationalities, and Peoples’ Regional State (SNNPRS), Ethiopia and has an average elevation of 1880 m. The area is inhabited by heterogeneous people of diverse ethnic groups, the majority of whom are Sidama people. The area is known for its cash crops including sugarcane and *khat* (*Catha edulis*). *Enset* (*Ensete ventricosum*), and maize are major food crops in the area. The Wondo Genet Forestry plantation is prominent in the area with several small-scale irrigation canals that irrigate various crop farms. The area has been known for its endemicity of malaria and helminth infections [11].

### Study population and sample collection

The cross-sectional study was conducted from December 2009 to July 2010 for over eight months. The area is characterized by seasonal malaria transmission and samples for both malaria detection and stool processing were collected from febrile malaria suspected patients visiting Kella, Aruma and Busa health centers serving seventeen ‘*kebeles’* (subdistricts - the lowest administrative units) of Wondo Genet District. The inclusion criteria of the study participants were febrile cases suspected for malaria, residing in the study area for more than a year and provision of informed consent by the patients or their legal guardians or parents for children younger than 18 years. Study participants having a febrile illness other than malaria, pregnant women and non-consenting were excluded from the study.

Sample size was determined using single proportion formula at 95% confidence interval (CI) level (Z = 1.96) with the expected prevalence of 50% for malaria and 5% marginal error. The sample size was then calculated as *n* = Z^2^ [P (1-P)]/d^2^, where: n = sample size, P = expected proportion in the study area, Z 1-α/2 = CI of 95%, d = marginal error to be tolerated. Accordingly, a total of 427 study participants were included in the study.

### Malaria parasite identification and determination of parasitaemia

Two slides were prepared for each febrile patient. The slides were free of dust, grease, soap, fingerprints and other debris so that the blood adheres to the slide for proper staining. On each slide, thick and methanol fixed thin blood films were prepared from capillary blood obtained by finger prick using disposable sterile lancet. Each blood smear was stained using 3% Giemsa stain for 30 min and examined according to WHO [21]. The stained slides were examined under a light microscope using 100x oil immersion objective. Up to 300 fields were examined before a blood smear was considered negative for the parasite. Giemsa-stained thin and thick blood films were used for the identification of *Plasmodium* species and determination of parasitaemia, respectively. Parasitaemia was calculated per 200 white blood cells assuming 8000 white blood cells per microliter of blood and classified as low, medium, high and hyperparasitaemia according to Garcia [22].

### Stool examination for intestinal helminth infections

Stool specimens were collected from the study participants using clean plastic vial and were processed for microscopic examination using Kato-Katz thick smear method (two slides for each specimen). Kato-Katz thick smears were prepared from stool specimen using 41.7 mg templates and examined quantitatively under 40x microscope objectives for ova of hookworms within 15–30 min. All the slides were carefully examined twice for the ova of other intestinal helminths after 30 min on the same day. The number of eggs per gram was calculated by multiplying the average number of eggs per slide by 24. The intensity of infections was classified as light, moderate and heavy [23].

### Determination of the level of anaemia

Haemoglobin assays were carried out on finger-prick blood samples collected with sterile disposable lancet and the reading was carried out using a portable haemoglobinometer (Hemocue Hb 201^+^ Ängelholm, Sweden) and anaemia was defined as per WHO criteria [24].

### Statistical analysis

SPSS version 17 (SPSS Inc., Chicago, IL) was used for the data entry and statistical analysis. Student’s *t* test and ANOVA were used to test for difference in proportions and means. Logistic regression analysis was used to determine the association between variables. Values were considered statistically significant when *P* values were less than 0.05 at 95% confidence interval (CI).

## Results

### Study participants

Overall, 427 malaria suspected febrile patients from three health centers of the study area were screened for malaria parasites and intestinal helminthiases between 2009 and 2010. Fifty five percent of the participants were males and 45% were females. The study participant’s age ranged from 6 to 77 years (mean ± SD = 20.8 ± 11.7) (Table 1).

**Table 1:** Prevalence of malaria, helminthiases and malaria-helminthiases coinfections among the study participants by sex and age category, Wondo Genet, southern Ethiopia, 2009–2010

Variable	Gender			Age category (years)		
	Male	Female	Total	5 to 14	> 14	Total
Malaria infection *N* (%)	154 (65.25)	122 (63.87)	276 (64.64)	98 (64.90)	178 (64.49)	276 (64.64)
Helminthiases *N* (%)	110 (46.61)	86 (45.03)	196 (45.90)	74 (49.01)	122 (44.20)	196 (45.90)
Coinfection *N* (%)	83 (19.43)	62 (14.52)	145 (33.96)	54 (12.64)	91 (21.31)	145 (33.96)
Total Examined *N* (%)	236 (55.3)	191 (44.7)	427 (100)	151 (35.4)	276 (64.64)	427 (100)

### Prevalence of malaria infection and the status of anaemia

Out of a total 427 study participants screened for malaria, 276 (64.64%) were positive for malaria parasites. The *Plasmodium* species detected were *P. falciparum* 202 (47.31%), *P. vivax* 71 (16.62%) and mixed infection by both *Plasmodium* species 3 (0.70%). There were no severe malaria cases presented during the study period.

The prevalence of anaemia was 43.12% with the mean haemoglobin level of 12.38 ± 1.89 g/dL. About 48.78% of *P. falciparum* and 39.44% of *P. vivax* infected study participants were anaemic with the mean haemoglobin level of 12.15 ± 1.98 g/dL and 12.48 ± 1.43 g/dL, respectively (range: 7.0 to 18.5 g/dL). There was no severe anaemia (haemoglobin < 5 g/dL) among the study participants. Among the *P. falciparum* infected study participants aged between five to < 12 years, 53.57% were anaemic (mean haemoglobin = 11.46 ± 1.98 g/dL). Likewise, 78.26% of children aged between 12 and 15, 29.63% of non-pregnant women age > 15 year old and 50.00% of males age greater than 15 years were found to be anaemic. There was statistically significant difference between the haemoglobin concentration of anaemic and non-anaemic *P. falciparum* infected study participants (*t*-test = 18.00; *P* < 0.001) (Table 2).

**Table 2:** Anaemia among malaria infected study participants by age group, Wondo Genet, southern Ethiopia, 2009–2010

Age group of *P. falciparum* infected participants	Haemoglobin (g/dL), Mean (SD)	Status of anaemia				*t*-test	*P* value
		Anaemic		Non-anaemic			
		*n* (%)	mean (SD)	*n* (%)	mean (SD)		
5- < 12, children (*n* = 56)	11.46 (1.98)	30 (53.57)	9.91 (0.92)	26 (46.43)	26, 13.25 (1.18)	11.87	< 0.001
12–15, children (*n* = 23)	10.89 (1.47)	18 (78.26)	10.26 (0.85)	5 (21.74)	13.14 (0.80)	6.74	< 0.001
> 15, non-pregnant women (*n* = 54)	12.46 (1.73)	16 (29.63)	10.31 (0.94)	38 (70.37)	13.36 (1.05)	10.03	< 0.001
> 15, men (*n* = 72)	12.87 (1.95)	36 (50.00)	11.39 (1.15)	36 (50.00)	14.34 (1.36)	9.94	< 0.001
Total = 205	12.15 (1.98)	100 (48.78)	10.57 (1.18)	105 (51.22)	13.66 (1.28)	18.00	< 0.001
*P. vivax* infected participants (*n* = 71)	12.48 (1.43)	28 (39.44)	11.21 (0.99)	43 (60.56)	13.30 (1.02)	8.54	< 0.001

### Intensity of helminth infections

Out of the 427 study participants, 196 (45.90%) were positive for at least one helminth infection. Intestinal helminths observed were *Ascaris lumbricoides* (33.96%), *Trichuris trichiura* (21.55%), *Schistosoma mansoni* (13.35%), hookworms (6.79%) and *Hymenolepis nana* (1.41%). According to the WHO [23] classes of intensity, *S. mansoni* had 248.8 EPG whereas the mean intensity of *T. trichiura*, *A. lumbricoides*, hookworms and *H. nana* infections were 214.4, 2,003.6, 145.7 and 584 EPG, respectively.

### Correlation of helminth coinfection with malaria infection

The univariate and multivariate analysis for the association of gender, age or presence/absence of helminthiases coinfection on the course of malaria infection was presented in Table 3. Accordingly, gender and age had no association with the occurrence of malaria; whereas, the presence of helminth coinfection had a positive association (AOR = 2.17, 95% CI: 1.44–3.28; *P* < 0.001).

**Table 3:** Association of gender, age (years) and helminthiases status with malaria among the study participants, Wondo Genet, southern Ethiopia, 2009–2010

Variable	Malaria infection		Crude OR (95% CI)	*P* value	AOR (95% CI)	*P* value
	Positive (%)	Negative (%)				
Gender						
Females	122 (63.87)	69 (36.13)	1.00^a^		1.00^a^	
Males	154 (65.25)	82 (34.75)	1.06 (0.71–1.58)	0.767	1.05 (0.18–1.66)	0.812
Age						
5–14 yrs	98 (64.90)	53 (35.10)	1.00^a^		1.00^a^	
> 14 yrs	178 (64.49)	98 (35.51)	0.98 (0.65–1.49)	0.933	1.02 (0.67–1.55)	0.938
Helminth infection						
Uninfected	131 (56.71)	100 (43.29)	1.00^a^		1.00^a^	
Infected	145 (73.98)	51 (26.02)	2.17 (1.44–3.28)	< 0.001	2.17 (1.44–3.28)	< 0.001

Note: ^a^Reference; *AOR* Adjusted odds ratio

### The odds of helminths coinfection on the occurrence of malaria, status of malaria associated anaemia and parasitaemia

In the present study, it was observed that helminth coinfection had no significant association with the level of haemoglobin concentration among malaria positive study participants. However, from a single helminth coinfection analysis among malaria infected participants, *Schistosoma mansoni* had increased the odds of developing anaemia (OR = 14.4, 95% CI: 1.37–150.80; *P* = 0.026) (Table 4). However, there was no correlation with helminth coinfection and the level of parasitaemia due to *P. falciparum* infection (Fischer’s exact-value = 4.48; *P* = 0.217, 95% CI). The Multivariable logistic regression analysis with single helminth coinfection with malaria showed that *Schistosoma mansoni* (AOR = 1.96, 95% CI: 1.20–3.21) and *Ascaris lumbricoides* (AOR = 2.58, 95% CI: 1.22–5.43) had positive associations with the occurrence of malaria infection.

**Table 4:** Association of helminths coinfections on the occurrence of anaemia among malaria infected study participants, Wondo Genet, southern Ethiopia, 2009–2010

Variable	Total	Anaemia (%)	Normal (%)	OR (95% CI)	*P*-value
Coinfection	145	64 (44.14)	81 (55.86)		
Malaria/Hook worm	0	0 (0.00)	0 (0.00)		
Malaria/*Trichuris trichuria*	13	5 (38.46)	8 (61.54)	1.00^a^	
Malaria/*Ascaris lumbricoides*	52	27 (51.92)	25 (48.08)	1.73 (0.50–5.99)	0.388
Malaria/*Schistosoma mansoni*	10	9 (90.00)	1 (10.00)	14.4 (1.37–150.80)	0.026
Malaria/*Hymenolepis nana*	0	0 (0.00)	0 (0.00)		
Malaria/Any single helminth Infection	75	41 (54.67)	34 (45.33)	1.93 (0.58–6.45)	0.286
Malaria/multiple helminth Infection	70	23 (32.86)	47 (67.14)	0.78 (0.23–2.66)	0.695

Note: ^a^Reference

## Discussion

Malaria and helminths coinfection is a serious public health problem in developing countries where their distribution overlaps [1, 2, 15, 25]. Wondo Genet is one of such places in Ethiopia where malaria and helminth parasites transmission overlaps and poses a major public health problem [11, 26]. This is partly attributable to small scale irrigation-based agricultural activities in the area that maintain year-round transmission of malaria and helminthiases. Irrigation provides favorable environment that would support the life cycle of the malaria parasite vectors, the intermediate hosts of Schistosome parasites and prevents destruction of soil transmitted helminth parasites. The prevalence of helminth-malaria parasite coinfection in this study (33.96%) was relatively lower than that reported in a previous study in the study area (67%) [11], Halaba Kulito district in southern Ethiopia (55.7%) [25] and northwest Ethiopia (53.9%) [27]. This may be an indication of the impact of malaria control measures which included case-management with drug treatment, application of indoor residual spraying (IRS), distribution of insecticide-treated nets (ITN) and integrated vector management measures in use at the time of the study [28].

Similar to previous reports on *A. lumbricoides*, *T. trichiura* and *S. mansoni* in different parts of Ethiopia [8, 11, 26], the present studies showed that these helminths are the dominant helminth parasites in the country. The prevalence of hookworm infection (6.79%) observed in the present study is lower than that of the national prevalence (16%) [29]. Similarly, the prevalence of *A. lumbricoides* (33.96%) and *T. trichiura* (21.55%) were lower than those of the national prevalence 37 and 30%, respectively [29]. Intestinal helminth infections were reported to have varied prevalence in different part of the country, 83.3% in Langano area [30], 82.4% in Zarima town [31] and 43.5% in Butajira [32]. Similarly, this study reported the total helminthiases burden of 45.90% among the study participants. This site-specific heterogeneity might be related to differences in target population, socioeconomic status, cultural inclination, level of sanitation practices and ecoepidemiological variations [32–34].

Although the causes of anaemia are known to be complex and multi-factorial, it is a major complication of malaria [35]. An inadequate intake of iron is another important factor in iron deficiency anaemia [36]. In malaria endemic areas of Africa, an overall prevalence of anaemia was reported to range from 49 to 89% mainly among children [37]. Hence, the prevalence of anaemia due to *P. falciparum* among children aged five to < 12 years (53.57%) and those aged 12–15 years (78.26%), reported in the present study, falls within this range. Nutritional deficiency and other etiologies might have added to this burden of anaemia.

Helminth parasite coinfection, especially *S. mansoni* and *A. lumbricoides,* was found to be positively associated with malaria infection. This finding is similar with a report from Dore Bafeno, southern Ethiopia [10] and from Osogbo, Nigeria [38]. However, concurrent intestinal helminthiases did not have profound association with the severity of parasitaemia due to *P. falciparum* infection. Other reports from the country showed the opposite finding in that helminth coinfections resulted in higher mean level of parasitaemia [11] and increased malaria parasite density [8].

Based on the information that malaria and intestinal helminth coinfections reduce haemoglobin levels, it is presumable that their combined presence might interact to enhance the risk of anaemia. Several reports from Kenya, Nigeria, Thailand and some other countries of Africa have also suggested the existence of an additive impact of helminth-malaria parasite coinfection on anaemia [16, 39–41]. In the present study, *S. mansoni* was found to increase the odds of developing anaemia. Schistosome coinfection might be exacerbating anaemia arising from malaria [42]. This is corroborated by the report by Getie and co-investigators from Northwest Ethiopia [43]. Other report claimed that *S. mansoni* coinfection might reduce the effectiveness of malaria treatment [44] indicating the need for integrated prevention approach.

The present study showed the occurrence of helminth parasite coinfections with malaria and its association with the occurrence of malaria infection and the development of anaemia. The finding has relevant implications for control interventions of malaria and helminth parasite infections. However, it was found difficult to reach at a definitive conclusion about the interaction of helminthiases and malaria based on the cross-sectional studies conducted. This is the limitation of the study and a longitudinal/cohort study design on a mixed infection model would be required for a better understanding of the effects of helminth-malaria parasite coinfections.

## Conclusions

Malaria and helminthiases are important public health problems in Wondo Genet. Lower prevalence of malaria compared with that of previous report [11] is attributable to the ongoing malaria intervention program. However, a comprehensive monitoring and evaluation of the intervention programs in the study area against both parasite groups will be required. The role of helminth parasite coinfection on the severe forms of malaria, and on treatment outcomes should be investigated as this would help design effective control approaches. A longitudinal/cohort study design on a mixed infection model would be required for a better understanding of the effect helminth-malaria parasite coinfections. Data on the role of helminth coinfections on the immunomodulation, and pathophysiology of malaria need to be generated.

## Data Availability

The datasets used and/or analysed during the current study are available from the corresponding author on reasonable request.

## Abbreviations

AOR: Adjusted odds ratio
SNNPRS: Southern Nations, Nationalities, and Peoples’ Regional State
WHO: World Health Organisation

## References

[1] Kinung'hi SM, Magnussen P, Kaatano GM, Kishamawe C, Vennervald BJ (2014). Malaria and helminth co-infections in school and preschool children: a cross-sectional study in Magu District, North-Western Tanzania. PLoS One.

[2] De Silva NR, Brooker S, Hotez PJ, Montresor A, Engels D, Savioli L (2003). Soil-transmitted helminth infections: updating the global picture. Trends Parasitol.

[3] WHO. World malaria report 2016: summary: World Health Organization (WHO); 2017.

[4] WHO. Schistosomiasis. https://www.who.int/news-room/fact-sheets/detail/schistosomiasis. Accessed 5 Feb 2019.

[5] Njunda AL, Fon SG, Assob JCN, Nsagha DS, Kwenti TDB, Kwenti TE (2015). Coinfection with malaria and intestinal parasites, and its association with anaemia in children in Cameroon. Infect Dis Poverty.

[6] McArdle AJ, Turkova A, Cunnington AJ (2018). When do co-infections matter?. Curr Opin Infect Dis.

[7] Efunshile AM, Olawale T, Stensvold CR, Kurtzhals JAL, König B (2015). Epidemiological study of the association between malaria and helminth infections in Nigeria. Am J Trop Med Hyg..

[8] Degarege A, Animut A, Legesse M, Erko B (2009). Malaria severity status in patients with soil-transmitted helminth infections. Acta Trop.

[9] Nacher M, Singhasivanon P, Yimsamran S, Manibunyong W, Thanyavanich N, Wuthisen P, Looareesuwan S (2002). Intestinal helminth infections are associated with increased incidence of *Plasmodium falciparum* malaria in Thailand. J Parasitol.

[10] Degarege A, Legesse M, Medhin G, Animut A, Erko B (2012). Malaria and related outcomes in patients with intestinal helminths: a cross-sectional study. BMC Infect Dis.

[11] Mulu A, Legesse M, Erko B, Belyhun Y, Nugussie D, Shimelis T, Kassu A (2013). Epidemiological and clinical correlates of malaria-helminth co-infections in southern Ethiopia. Malaria J.

[12] Sokhna C, Le Hesran J-Y, Mbaye PA, Akiana J, Camara P, Diop M, Ly A (2004). Increase of malaria attacks among children presenting concomitant infection by *Schistosoma mansoni* in Senegal. Malaria J..

[13] Spiegel A, Tall A, Raphenon G, Trape J-F, Druilhe P (2003). Increased frequency of malaria attacks in subjects co-infected by intestinal worms and *Plasmodium falciparum* malaria. Trans R Soc Trop Med Hyg.

[14] Yatich Nelly J., Funkhouser Ellen, Ehiri John E., Agbenyega Tsiri, Stiles Jonathan K., Rayner Julian C., Turpin Archer, Ellis William O., Jiang Yi, Williams Jonathan H., Afriyie-Gwayu Evans, Phillips Timothy, Jolly Pauline E. (2010). Malaria, Intestinal Helminths and Other Risk Factors for Stillbirth in Ghana. Infectious Diseases in Obstetrics and Gynecology.

[15] Matangila JR, Doua JY, Linsuke S, Madinga J, Inocêncio da Luz R, Van Geertruyden J-P, Lutumba P (2014). Malaria, schistosomiasis and soil transmitted helminth burden and their correlation with anemia in children attending primary schools in Kinshasa, Democratic Republic of Congo. PloS One.

[16] Nacher M, Singhasivanon P, Gay F, Phumratanaprapin W, Silachamroon U, Looareesuwan S (2001). Association of helminth infection with decreased reticulocyte counts and hemoglobin concentration in Thai falciparum malaria. Am J Trop Med Hyg..

[17] Abanyie FA, McCracken C, Kirwan P, Molloy SF, Asaolu SO, Holland CV, Gutman J (2013). Ascaris co-infection does not alter malaria-induced anaemia in a cohort of Nigerian preschool children. Malaria J..

[18] Nacher M, Singhasivanon P, Silachamroon U, Treeprasertsuk S, Vannaphan S, Traore B, Gay F (2001). Helminth infections are associated with protection from malaria-related acute renal failure and jaundice in Thailand. Am J Trop Med Hyg.

[19] Nacher M, Gay F, Singhasivanon P, Krudsood S, Treeprasertsuk S, Mazier D, Vouldoukis I (2000). *Ascaris lumbricoides* infection is associated with protection from cerebral malaria. Parasite Immunol.

[20] Shapiro AE, Tukahebwa EM, Kasten J, Clarke SE, Magnussen P, Olsen A, Kabatereine NB (2005). Epidemiology of helminth infections and their relationship to clinical malaria in Southwest Uganda. Trans R Soc Trop Med Hyg.

[21] WHO (1991). Basic laboratory methods in medical parasitology.

[22] Garcia LS (2001). Diagnostic Medical Parasitology.

[23] WHO: Prevention and control of schistosomiasis and soil-transmitted helminthiasis. World Health Organization (WHO) technical report series by Expert Committee*.* 2002. 12592987

[24] WHO. Worldwide prevalence of anaemia 1993–2005: World Health Organization (WHO) global database on anaemia. 2008.

[25] Degarege A, Animut A, Legesse M, Erko B (2010). Malaria and helminth co-infections in outpatients of Alaba Kulito health center, southern Ethiopia: a cross sectional study. BMC Res Notes..

[26] Erko B, Medhin G (2003). Human helminthiasis in Wondo Genet, southern Ethiopia, with emphasis on geohelminthiasis. Ethiop Med J.

[27] Alemu A, Shiferaw Y, Ambachew A, Hamid H (2012). Malaria helminth co–infections and their contribution for aneamia in febrile patients attending Azzezo health center, Gondar, Northwest Ethiopia: a cross sectional study. Asian Pac J Trop Med.

[28] Federal Ministry of Health: National strategic plan for malaria prevention, control and elimination in Ethiopia 2010-2015. Federal ministry of health (FMOH), Federal Democratic Republic of Ethiopia, Addis Ababa 2009.

[29] Tadesse Z, Hailemariam A, Kolaczinski JH (2008). Potential for integrated control of neglected tropical diseases in Ethiopia. Trans R Soc Trop Med Hyg.

[30] Legesse M, Erko B (2004). Prevalence of intestinal parasites among schoolchildren in a rural area close to the southeast of Lake Langano. Ethiopia Ethiop J Health Dev.

[31] Alemu A, Atnafu A, Addis Z, Shiferaw Y, Teklu T, Mathewos B, Birhan W (2011). Soil transmitted helminths and *Schistosoma mansoni* infections among school children in Zarima town, Northwest Ethiopia. BMC Infect Dis.

[32] Belyhun Y, Medhin G, Amberbir A, Erko B, Hanlon C, Alem A, Venn A (2010). Prevalence and risk factors for soil-transmitted helminth infection in mothers and their infants in Butajira, Ethiopia: a population based study. BMC Public Health.

[33] Alelign Tilahun, Degarege Abraham, Erko Berhanu (2015). Soil-Transmitted Helminth Infections and Associated Risk Factors among Schoolchildren in Durbete Town, Northwestern Ethiopia. Journal of Parasitology Research.

[34] Getachew M, Tafess K, Zeynudin A, Yewhalaw D (2013). Prevalence soil transmitted helminthiasis and malaria co-infection among pregnant women and risk factors in Gilgel gibe dam area, Southwest Ethiopia. BMC Res Notes.

[35] Newton C, Warn P, Winstanley P, Peshu N, Snow R, Pasvol G, Marsh K (1997). Severe anaemia in children living in a malaria endemic area of Kenya. Tropical Med Int Health.

[36] Tatala S, Svanberg U, Mduma B (1998). Low dietary iron availability is a major cause of anemia: a nutrition survey in the Lindi District of Tanzania. Am J Clin Nutr.

[37] WHO. Estimates of maternal mortality: a new approach by World Health Organization (WHO) and UNICED. Geneva; 1996.

[38] Ojurongbe O, Adegbayi AM, Bolaji OS, Akindele AA, Adefioye OA, Adeyeba OA (2011). Asymptomatic falciparum malaria and intestinal helminths co-infection among school children in Osogbo. Nigeria J Res Med Sci.

[39] Akhwale WS, Lum JK, Kaneko A, Eto H, Obonyo C, Björkman A, Kobayakawa T (2004). Anemia and malaria at different altitudes in the western highlands of Kenya. Acta Trop.

[40] Egwunyenga A, Ajayi J, Nmorsi O, Duhlinska-Popova D (2001). Plasmodium/intestinal helminth co-infections among pregnant Nigerian women. Mem Inst Oswaldo Cruz.

[41] Brooker S, Clements A, Hotez P, Hay S, Tatem A, Bundy D, Snow R (2006). The co-distribution of *Plasmodium falciparum* and hookworm among African schoolchildren. Malaria J..

[42] Friedman JF, Kanzaria HK, McGarvey ST (2005). Human schistosomiasis and anemia: the relationship and potential mechanisms. Trends Parasitol.

[43] Getie S, Wondimeneh Y, Getnet G, Workineh M, Worku L, Kassu A, Moges B (2015). Prevalence and clinical correlates of *Schistosoma mansoni* co-infection among malaria infected patients. Northwest Ethiopia BMC Res Notes.

[44] Mbah MLN, Skrip L, Greenhalgh S, Hotez P, Galvani AP (2014). Impact of *Schistosoma mansoni* on malaria transmission in sub-Saharan Africa. PLoS Negl Trop Dis.

